# Nonlinear Association Between Atherogenic Index of Plasma and Unstable Carotid Plaque: A Single-Center Retrospective Study

**DOI:** 10.3390/jcdd12110443

**Published:** 2025-11-11

**Authors:** Guijun Huo, Yao Tang, Dayong Zhou

**Affiliations:** The Affiliated Suzhou Hospital of Nanjing Medical University, Suzhou Municipal Hospital, Suzhou 215002, China; huoguijun2012@163.com

**Keywords:** atherogenic index of plasma, carotid atherosclerosis, unstable carotid plaque

## Abstract

Background: The atherogenic index of plasma (AIP) is a marker of lipids and atherosclerosis. However, the association between the AIP and the risk of unstable carotid plaque remains unclear. Methods: A total of 10,732 patients were enrolled. Carotid ultrasound was used to assess the stability of carotid plaque. The AIP was calculated using the formula AIP = log (TG/HDL-C). Logistic regression was used to investigate the association between the AIP and unstable carotid plaque. The nonlinear association between the AIP and unstable carotid plaque was analyzed using restricted cubic splines (RCSs), and a two-segment logistic regression risk model was constructed for both sides of the inflection point. Results: Of the total number of patients, 7834 had stable carotid plaque and 2898 had unstable carotid plaque. Our findings demonstrated an inverted U-shaped association between the AIP and the risk of unstable carotid plaque (threshold = 0.10). When the AIP was <0.10, a significant positive association with unstable carotid plaque emerged (OR: 1.24, 95% CI: 1.07, 1.44); when the AIP was ≥0.10, it showed a significant negative association with unstable carotid plaque (OR: 0.78, 95% CI: 0.70, 0.88). Conclusions: Our findings demonstrated an inverted U-shaped association between the AIP and the risk of unstable carotid plaque, with a threshold of 0.10. The AIP could serve as a potential biomarker for unstable carotid plaque risk assessment.

## 1. Introduction

Carotid plaque is the local manifestation of atherosclerosis on the carotid artery wall, which is mainly formed by lipid deposition, inflammatory cell infiltration, smooth muscle cell proliferation, and fibrous tissue proliferation [1]. As plaques progress, they can lead to vascular stenosis, restricted blood flow, and even plaque rupture causing thrombosis, which is one of the important risk factors for ischemic stroke [2,3,4,5]. Research has shown that about 7–18% of ischemic strokes are associated with carotid plaques, especially unstable carotid plaques, which are more prone to rupture and lead to stroke events due to their thin fibrous cap, large lipid core, and active inflammation [3,6,7,8,9,10,11]. Therefore, early identification and intervention of unstable carotid plaques are crucial for preventing stroke.

Dyslipidemia is characterized by lipid metabolism disorder, and the abnormal levels of triglycerides (TG), total cholesterol (TC), high-density lipoprotein cholesterol (HDL-C), and low-density lipoprotein cholesterol (LDL-C) are believed to be closely related to the development of atherosclerosis [12,13,14]. The atherogenic index of plasma (AIP) is a new lipid marker proposed by Dobiasova and Frohlich in 2001 [15]. The AIP effectively integrates TG and HDL-C levels, which is not only an indicator of dyslipidemia, but also a reliable predictor of atherosclerosis [16,17]. Furthermore, recent research has shown that the AIP demonstrates superior predictive value for atherosclerosis compared to traditional lipid parameters [18,19]. Additionally, a cohort study from China suggests that an increase in AIP levels is associated with a higher incidence of carotid plaque, which can serve as a potential biomarker for carotid plaque risk assessment [20]. However, the association between the AIP and the risk of unstable carotid plaque remains unclear.

To address these knowledge gaps, we conducted a retrospective study to explore the association between the AIP and the risk of unstable carotid plaque, and provide more evidence on the practical application of the AIP in the real world. Given that prior studies have shown potential threshold or saturation effects between lipid-related indices and atherosclerotic outcomes, we hypothesized a priori that the association between the AIP and unstable carotid plaque might be nonlinear. To capture this potential complexity, restricted cubic spline (RCS) modeling was used to flexibly characterize the dose–response relationship.

## 2. Methods

### 2.1. Study Population

**Figure 1:**
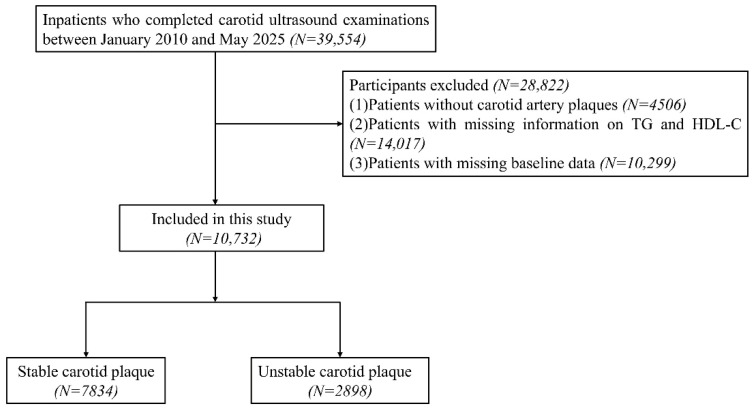
Flow chart of patient recruitment.

This study was designed as a retrospective cross-sectional single-center study conducted at The Affiliated Suzhou Hospital of Nanjing Medical University. The patients screening process for this study is presented in Figure 1. This retrospective study included 39,554 inpatients who underwent carotid artery ultrasonography from January 2010 to May 2025 in The Affiliated Suzhou Hospital of Nanjing Medical University. We excluded 4506 patients without carotid artery plaques. Additionally, 14,017 patients were removed due to missing information on TG and HDL-C. Furthermore, 10,299 patients were excluded due to lack of baseline information. Consequently, 28,822 patients were excluded, resulting in a final cohort of 10,732 patients for this study. We confirm that all research was performed in accordance with relevant guidelines/regulations.

### 2.2. Data Collection and Definition

Fasting blood samples were taken from all patients and biochemical measurements were analyzed. The following clinical data were obtained, including sex, age, hypertension, dyslipidemia, diabetes, hypertension medications, diabetes medications, dyslipidemia medications, systolic blood pressure (SBP), diastolic blood pressure (DBP), height, weight, body mass index (BMI), fasting plasma glucose (FPG), total cholesterol (TC), triglyceride (TG), high-density lipoprotein cholesterol (HDL-C), low-density lipoprotein cholesterol (LDL-C), and HbA1c. Serum biochemical parameters were determined with a biochemical auto-analyzer (Hitachi7600, Tokyo, Japan). Blood routine parameters were determined with an automatic blood cell analyzer (BC-6800Plus, Mindray, Shenzhen, China). The AIP was obtained by using the following formula: AIP = log (TG/HDL-C).

### 2.3. Assessment of Stability of Carotid Plaque

Carotid ultrasound is performed by certified professional technicians using an ultrasound diagnostic system (Resona7, Mindray, China). The common carotid artery, internal carotid artery and carotid bifurcation were scanned, respectively. Stable carotid plaque has uniform texture, smooth and regular surface, and high level or even plaque echo. Unstable carotid plaque was defined as low level or heterogeneous echogenic plaque according to plaque echo density, and as incomplete fibrous cap plaque or ulcerative plaque according to plaque morphology [21,22]. Carotid artery ultrasound results are reviewed by two independent technicians, and a consistent result is obtained. When the results of two technicians are inconsistent, a third technician is sought for consistent verification, and the consistent result shall prevail.

### 2.4. Statistical Analysis

Normally distributed continuous data were presented as mean ± standard deviation and analyzed for statistical significance using a one-way ANOVA. Non-normally distributed continuous data were expressed as median and interquartile range and analyzed by the Kruskal–Wallis test. Categorical variables are represented as counts and percentages and were evaluated using the Chi-squared test.

Logistic regression models were utilized, which calculated odds ratios (ORs) along with 95% confidence intervals (95%CIs). Three models with varying levels of covariate adjustment were developed. Model 1 had no adjustments, Model 2 adjusted for sex, age, hypertension, dyslipidemia, and diabetes, and Model 3 further adjusted for hypertension medications, diabetes medications, dyslipidemia medications, SBP, DBP, BMI, FPG, TC, LDL-C, and HbA1c. Furthermore, a restricted cubic spline (RCS) model with four knots located at the 5th, 35th, 65th, and 95th percentiles of AIP distribution was used to flexibly explore potential nonlinear relationships (*p* < 0.05). If the association exhibited nonlinearity, the threshold value was estimated by trying all possible values, choosing the threshold point with the highest likelihood. Subsequently, a two-piecewise logistic regression model was employed on both sides of the inflection point to investigate the association between the AIP and the risk of unstable carotid plaque.

To improve the credibility of our research results, we conducted a sensitivity analysis. We retained 10,299 patients who lacked baseline information for reanalysis. All statistical analyses were conducted using R version 4.2.2, with *p*-values < 0.05, which was considered statistically significant.

## 3. Results

### 3.1. Population Characteristics

The study included 10,732 patients, with a sex distribution of 5900 (55.0%) men and 4832 (45.0%) women. The average age of patients was 71.13 ± 10.70 years, and 2898 (27.0%) patients with unstable carotid plaque. Individuals in the unstable carotid plaque group are generally men, older, and tended to have diabetes and dyslipidemia. Additionally, the group with unstable carotid plaque had a higher proportion of patients using hypertension and dyslipidemia medications. Furthermore, compared to those with stable carotid plaque, patients with unstable carotid plaque had significantly lower levels of DBP, BMI, FPG, TC, TG, and the AIP. The demographic and clinical characteristics of all patients were shown in Table 1.

### 3.2. Association Between AIP and Unstable Carotid Plaque

The association between the AIP and unstable carotid plaque was explored using logistic regression models. As shown in Table 2, the unadjusted logistic analysis demonstrated (Model 1) that each 1-unit increase in the AIP was associated with a 11% reduction in unstable carotid plaque risk (OR: 0.89, 95% CI: 0.83, 0.94). Additionally, the adjusted ORs (95% CIs) for Q3 and Q4 compared to Q1 were 0.88 (95% CI: 0.78, 0.99), and 0.82 (95% CI: 0.73, 0.93), respectively. However, after adjust the potential confounding variables (Model 3), we did not observe a significant association between the AIP and unstable carotid plaques, whether as a continuous variable (OR: 0.95, 95% CI: 0.89, 1.02) or categorical variable (OR: 0.97, 95% CI: 0.85, 1.11).

### 3.3. RCS Analysis

**Figure 2:**
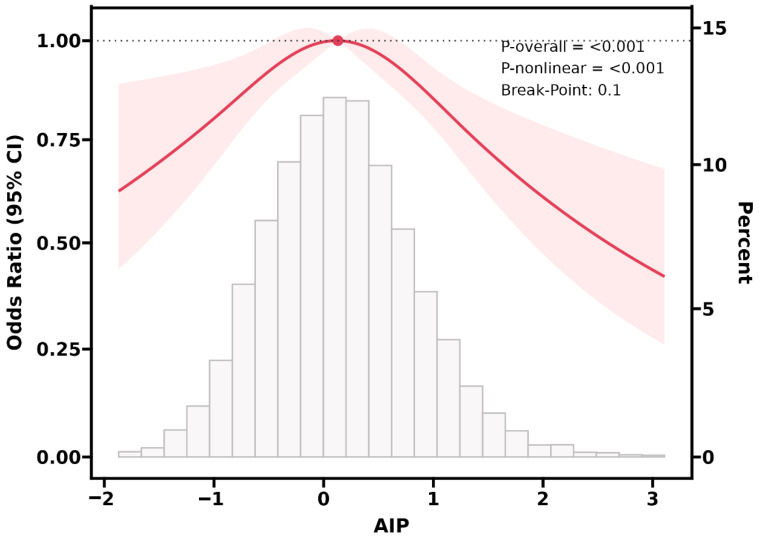
Restricted cubic spline regression analysis showed that there was a nonlinear relationship between the AIP and unstable carotid plaque. Adjusted for sex, age, hypertension, dyslipidemia, diabetes, hypertension medications, diabetes medications, dyslipidemia medications, SBP, DBP, BMI, FPG, TC, LDL-C, and HbA1c.

To further validate the association between the AIP and the risk of unstable carotid plaque, we employed RCS analysis. Our results demonstrated an inverted U-shaped association between the AIP and the risk of unstable carotid plaque (Figure 2). Additionally, we further fitted the association between the AIP and the risk of unstable carotid plaque using two segmented logistic regression model. Notably, when the AIP was <0.10, we observed that a 1-unit increase in the AIP was associated with a 24% increase in the risk of unstable carotid plaque (OR: 1.24, 95% CI: 1.07, 1.44). When the AIP was ≥0.10, each 1-unit increase in the AIP was associated with a 22% reduction in unstable carotid plaque risk (OR: 0.78, 95% CI: 0.70, 0.88) (Table 3).

### 3.4. Sensitivity Analysis

To assess the robustness of the findings, we conducted a sensitivity analyses. We retained 10,299 patients who lacked baseline information for reanalysis. The extent of missing data in this study is detailed in Table S1. To address potential bias, we employed multiple imputations to fill in the missing values. When we reanalyzed, there was no substantial change in the results. Specifically, after adjust the potential confounding variables (Model 3), we did not observe a significant association between the AIP and unstable carotid plaques, whether as a continuous variable (OR: 0.95, 95% CI: 0.89, 1.02) or categorical variable (OR: 0.99, 95% CI: 0.87, 1.14) (Table S2). RCS revealed a significant nonlinear relationship between the AIP and unstable carotid plaque risk, with a threshold of 0.10 (Figure S1). When the AIP was <0.10, we observed that a 1-unit increase in the AIP was associated with a 22% increase in the risk of unstable carotid plaque (OR: 1.22, 95% CI: 1.08, 1.37). When the AIP was ≥0.10, each 1-unit increase in the AIP was associated with a 23% reduction in unstable carotid plaque risk (OR: 0.77, 95% CI: 0.70, 0.85) (Table S3).

## 4. Discussion

In this retrospective study, we investigated the relationship between the AIP and the risk of unstable carotid plaque in the general population. We observed an inverted U-shaped association between the AIP and the risk of unstable carotid plaque. Additionally, threshold effect analysis revealed the inflection point of the AIP and unstable carotid plaque (threshold = 0.10). In conclusion, our study suggests that the AIP is a valuable marker of risk of unstable carotid plaque, which may help to advance unstable carotid plaque prevention measures.

Atherosclerosis, characterized by intimal lipid accumulation, fibrous tissue hyperplasia and calcinosis, has no obvious symptoms in the early stage and is the pathological basis of many cardiovascular diseases [23,24,25]. Previous studies have shown that carotid atherosclerosis patients have a significantly increased risk of cardiovascular disease, so to reduce the risk of cardiovascular disease, carotid atherosclerosis should be prioritized [26,27,28]. With the advancement of imaging technology, researchers not only focus on the degree of carotid artery stenosis, but also pay more attention to the composition and stability of carotid plaques. Previous studies have shown that unstable carotid plaques are closely associated with the risk of stroke [29,30,31,32]. Therefore, the systematic assessment of the stability of carotid plaques and the search for potential biomarkers are of great significance for the early identification and intervention strategy development of high-risk stroke populations.

Although these single biomarkers can predict the risk of unstable carotid plaque, but the predictive accuracy is still limited. Recent evidence suggests that using a combination of multiple biomarkers can provide a more comprehensive evaluation compared to a single biomarker [33,34,35,36]. The AIP, a novel and simple lipid parameter to assess the risk of atherosclerosis, is considered as a reliable predictor of atherosclerotic cardiovascular disease [15,37,38,39,40]. The research conducted by QU et al. demonstrates that elevated levels of theAIP are significantly correlated with an increased risk of stroke among middle-aged and elderly individuals [41]. The study by Liu et al., which included 1463 patients with acute ischemic stroke, suggests that the AIP may be an important independent predictor of functional impairment outcomes in patients with acute ischemic stroke [42]. A study involving 3468 healthy adult participants showed that a higher AIP was an independent predictor of increased arterial stiffness in healthy Korean men and women [43]. Min et al.’s study indicates that monitoring long-term AIP changes in middle-aged and elderly individuals with abnormal glucose metabolism can aid in the early identification of cardiovascular disease risk [44]. Additionally, a study utilizing the China Health and Retirement Longitudinal Study (CHARLS) framework has identified a significant and independent correlation between the AIP and the risk of developing diabetes [45]. Moreover, research from Chinese community population shows that the increase in the AIP level is related to the high incidence rate of carotid plaque, which can be used as a potential biomarker for risk assessment of carotid plaque [20]. However, most of the previous studies focused on atherosclerotic vascular diseases, while the stability of carotid plaque did not attract similar attention.

In our study, we included 10,732 patients and identified an inverted U-shaped nonlinear association between the AIP and the risk of unstable carotid plaque. Specifically, when the AIP is less than 0.10, we observed a positive association between the AIP and the risk of unstable carotid plaques. When the AIP exceeded 0.10, we observed a negative association between the AIP and the risk of unstable carotid plaques. Additionally, it is important to note that, in contrast to our research findings, Zhao et al.’s study, which involved 336 patients with acute ischemic stroke, did not find a significant association between the AIP and the risk of unstable carotid plaques [46]. Several factors may explain this discrepancy. Firstly, Zhao et al.’s study focused specifically on patients with ischemic stroke, which may not accurately represent the general population. Secondly, the sample size of 336 cases is relatively small. Furthermore, the patients were recruited from Qinghai Province in China, an area characterized by high altitude. These findings may be influenced by factors such as the heterogeneity of patient populations, the limited number of participants, and differences in geographic location and economic conditions. Our sensitivity analysis further confirms the nonlinear relationship between the AIP and the risk of unstable carotid plaques. Even with the inclusion of participants with missing data, a total of 21,031 patients were included, and this nonlinear association remained significant. These findings emphasize the necessity of evaluating the AIP as a primary prevention for unstable carotid plaques in clinical practice.

Therefore, to avoid the formation of unstable carotid plaques, it is necessary to maintain the AIP at an appropriate level.

The underlying mechanisms of the observed inverted U-shaped association between the AIP and unstable carotid plaque remain to be fully elucidated. Previous studies suggest that elevated AIP reflects insulin resistance, dyslipidemia, and proinflammatory metabolic states, which promote endothelial dysfunction and atherogenesis, thereby increasing the likelihood of plaque vulnerability [47,48,49,50,51]. The AIP is also positively correlated with systemic inflammatory and oxidative stress markers, which accelerate the degradation of the fibrous cap and intraplaque hemorrhage formation [52,53,54,55,56,57,58,59,60]. At higher AIP levels, the observed inverse relationship might reflect treatment-related or selection effects. Individuals with severe dyslipidemia often receive intensive lipid-lowering therapy, which can stabilize plaques and reduce inflammation. Moreover, high AIP levels may represent patients with more advanced or fibrotic plaques that are less prone to rupture, or those under closer medical monitoring (‘survivor bias’). These hypotheses require further validation in prospective and mechanistic studies [25,61,62].

Although this study revealed an inverted U-shaped relationship between the AIP and the risk of unstable carotid plaques, there are still many limitations. Firstly, this is a cross-sectional retrospective study; therefore, temporality and causality between the AIP and unstable carotid plaque cannot be established. Further prospective and mechanistic studies are warranted to validate our findings. Secondly, while multivariate adjustments were made to account for potential confounding factors, it is still possible that undiscovered confounders may influence the results. Thirdly, ultrasound may not be as accurate as computed tomography angiography (CTA) or magnetic resonance imaging (MRI) results in evaluating the stability of carotid plaque. However, it is undeniable that ultrasound is more widely used and popular today. Fourthly, due to the retrospective nature of this study, information for excluded patients was unavailable, which may introduce potential selection bias. However, sensitivity analyses including individuals with missing data yielded consistent results, suggesting that the exclusion of these patients did not materially affect our findings. Fifthly, although our models adjusted for the use of antihypertensive, antidiabetic, and dyslipidemia medications, detailed data on specific drug classes, treatment duration, and adherence were unavailable. In particular, statins and antiplatelet agents may influence both AIP levels and carotid plaque stability, leading to potential residual confounding. Future studies with more detailed medication records are warranted to clarify these effects. Finally, as this study was conducted in a single hospital in Suzhou, China, the findings may not be generalizable to other populations or ethnic groups. Multi-center studies across diverse regions are needed to validate the observed associations.

## 5. Conclusions

Our research results indicate that an inverted U-shaped relationship was observed between the AIP and the risk of unstable carotid plaque, with a threshold of 0.10. When the AIP < 0.10, it demonstrated a significant positive association with unstable carotid plaque risk; when the AIP ≥ 0.10, it demonstrated a significant negative association with unstable carotid plaque risk. Therefore, we advocate for monitoring the AIP as a potential biomarker for unstable carotid plaque risk assessment.

## Figures and Tables

**Table 1:** Patient demographics and baseline characteristics.

Characteristic	Overall	Stable Carotid Plaque	Unstable Carotid Plaque	*p*-Value
	N = 10,732	N = 7834	N = 2898	
Sex				<0.001
Men	5900 (55.0%)	4101 (52.3%)	1799 (62.1%)	
Women	4832 (45.0%)	3733 (47.7%)	1099 (37.9%)	
Age, year	71.13 ± 10.70	70.85 ± 10.90	71.87 ± 10.11	<0.001
Hypertension	8809 (82.1%)	6403 (81.7%)	2406 (83.0%)	0.122
Diabetes	7886 (73.5%)	5806 (74.1%)	2080 (71.8%)	0.015
Dyslipidemia	9871 (92.0%)	7096 (90.6%)	2775 (95.8%)	<0.001
Hypertension medications	7174 (66.8%)	5165 (65.9%)	2009 (69.3%)	<0.001
Diabetes medications	5806 (54.1%)	4331 (55.3%)	1475 (50.9%)	<0.001
Dyslipidemia medications	8615 (80.3%)	5986 (76.4%)	2629 (90.7%)	<0.001
SBP, mmHg	143.33 ± 22.80	143.46 ± 22.88	142.95 ± 22.55	0.298
DBP, mmHg	76.96 ± 17.04	77.14 ± 18.17	76.47 ± 13.50	0.040
Height, m	1.62 ± 0.19	1.62 ± 0.21	1.63 ± 0.14	0.023
Weight, kg	65.00 (56.50, 72.00)	65.00 (56.00, 72.00)	65.00 (57.00, 72.00)	0.444
BMI, kg/m^2^	24.03 (21.96, 26.30)	24.12 (22.03, 26.37)	23.88 (21.78, 26.13)	0.004
FPG, mmol/L	5.68 (4.94, 7.15)	5.73 (4.97, 7.21)	5.54 (4.87, 6.97)	<0.001
TC, mmol/L	4.31 ± 1.16	4.36 ± 1.16	4.18 ± 1.13	<0.001
TG, mmol/L	1.28 (0.90, 1.85)	1.31 (0.91, 1.88)	1.21 (0.87, 1.73)	<0.001
HDL-C, mmol/L	1.17 ± 0.31	1.17 ± 0.31	1.16 ± 0.31	0.094
LDL-C, mmol/L	2.73 ± 1.04	2.79 ± 1.04	2.59 ± 1.02	<0.001
HbA1c, %	7.19 ± 1.95	7.20 ± 1.97	7.15 ± 1.92	0.235
AIP	0.15 ± 0.69	0.17 ± 0.70	0.11 ± 0.67	<0.001

**Table 2:** Multivariable logistic regression analysis of the relationship between the AIP and the risk of unstable carotid plaque.

Characteristic	Event, *n*	Model 1			Model 2			Model 3		
		OR	95%CI	*p*	OR	95%CI	*p*	OR	95%CI	*p*
AIP (per 1 unit)	2898	0.89	0.83, 0.94	<0.001	0.85	0.80, 0.91	<0.001	0.95	0.89, 1.02	0.162
AIP quartile										
Q1	768	Ref			Ref			Ref		
Q2	765	0.99	0.88, 1.12	0.914	0.96	0.85, 1.08	0.473	1.10	0.97, 1.24	0.140
Q3	699	0.88	0.78, 0.99	0.033	0.82	0.73, 0.93	0.002	1.01	0.89, 1.15	0.868
Q4	666	0.82	0.73, 0.93	0.002	0.76	0.67, 0.87	<0.001	0.97	0.85, 1.11	0.679
*p* for trend				<0.001			<0.001			0.425

OR, odds ratio; CI, confidence interval. Model 1: unadjusted for any covariates. Model 2: adjusted for sex, age, hypertension, dyslipidemia, and diabetes. Model 3: adjusted for sex, age, hypertension, dyslipidemia, diabetes, hypertension medications, diabetes medications, dyslipidemia medications, SBP, DBP, BMI, FPG, TC, LDL-C, and HbA1c.

**Table 3:** Result of the two-piecewise logistic regression model.

Characteristic	Case/Total	OR	95%CI	*p*
Unstable carotid plaque				
Total	2898/10,732	0.95	0.89, 1.02	0.162
The inflection points of the AIP		0.10		
<0.10	1476/5164	1.24	1.07, 1.44	0.004
≥0.10	1422/5568	0.78	0.70, 0.88	<0.001
Log likelihood ratio				<0.001

OR, odds ratio; CI, confidence interval. Adjusted for sex, age, hypertension, dyslipidemia, diabetes, hypertension medications, diabetes medications, dyslipidemia medications, SBP, DBP, BMI, FPG, TC, LDL-C, and HbA1c.

## Data Availability

The datasets used and analyzed in the study are available from the corresponding author upon reasonable request.

## Supplementary Materials

The following supporting information can be downloaded at: https://www.mdpi.com/article/10.3390/jcdd12110443/s1, Figure S1: Restricted cubic spline regression analysis showed that there was a nonlinear relationship between AIP and unstable carotid plaque after retained 10299 patients who lacked baseline information.; Table S1: Distribution of variables with missing data. Table S2: Multivariable logistic regression analysis of the relationship between AIP and the risk of unstable carotid plaque after retained 10299 patients who lacked baseline information. Table S3: Result of the two-piecewise logistic regression model after retained 10299 patients who lacked baseline information.
