# Supplementary material for: Nonlinear Association Between Atherogenic Index of Plasma and Unstable Carotid Plaque: A Single-Center Retrospective Study
Source: J Cardiovasc Dev Dis. 2025 Nov 11;12(11):443. doi: 10.3390/jcdd12110443 (PMC12653747; doi:10.3390/jcdd12110443)
Supplement: Supplementary file 1 [file jcdd-12-00443-s001.zip › jcdd-3926416-supplementary.pdf]

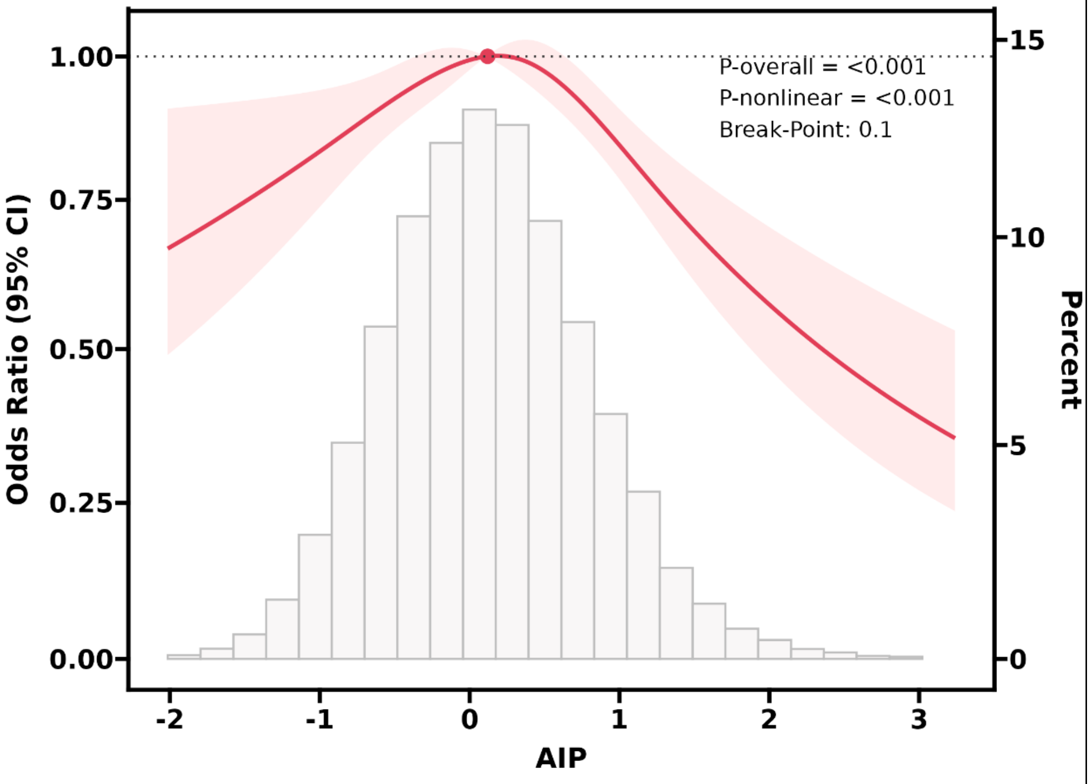

Fig. S1: Restricted cubic spline regression analysis showed that there was a nonlinear relationship between AIP and unstable carotid plaque after retained 10299 patients who lacked baseline information. Adjusted for sex, age, hypertension, dyslipidemia, diabetes, hypertension medications, diabetes medications, dyslipidemia medications, SBP, DBP, BMI, FPG, TC, LDL-C, and HbA1c.

Table S1 Distribution of variables with missing data

**Variables**	**Number of Missing**	**Missing proportion**
Height	6007	28.6%
Weight	6684	31.8%
BMI	6703	31.9%
SBP	6000	28.5%
DBP	6000	28.5%
FPG	1783	8.5%
HbA1c	4531	11.5%

Table S2 Multivariable logistic regression analysis of the relationship between AIP and the risk of unstable carotid plaque after retained 10299 patients who lacked baseline information.

**Characteristic**	**Event, n**	**Model 1**			**Model 2**			**Model 3**		
		**OR**	**95%CI**	**p**	**OR**	**95%CI**	**p**	**OR**	**95%CI**	**p**
AIP (per 1 unit)	4,673	0.93	0.88, 0.97	0.002	0.92	0.88, 0.97	0.002	0.95	0.89, 1.02	0.261
AIP quartile										

Q1	1,196	Ref			Ref			Ref		
Q2	1,236	1.04	0.95, 1.14	0.366	1.04	0.95, 1.14	0.379	1.11	0.98, 1.26	0.092
Q3	1,149	0.95	0.87, 1.04	0.262	0.95	0.87, 1.04	0.268	1.02	0.89, 1.16	0.784
Q4	1,092	0.89	0.81, 0.98	0.013	0.89	0.81, 0.98	0.013	0.99	0.87, 1.14	0.940
P for trend				0.003			0.003			0.591

OR, odds ratio; CI, confidence interval.

Model 1: unadjusted for any covariates.

Model 2: adjusted for sex, age, hypertension, dyslipidemia, and diabetes.

Model 3: adjusted for sex, age, hypertension, dyslipidemia, diabetes, hypertension medications, diabetes medications, dyslipidemia medications, SBP, DBP, BMI, FPG, TC, LDL-C, and HbA1c.

Table S3 Result of the two-piecewise logistic regression model after retained 10299 patients who lacked baseline information.

**Characteristic**	**Case/total**	**OR**	**95%CI**	**P**
**Unstable carotid plaque**				
Total	4673/21031	0.95	0.89, 1.02	0.261
The inflection points of AIP		0.10		
< 0.10	2406/10422	1.22	1.08, 1.37	0.001
≥ 0.10	2267/10609	0.77	0.70, 0.85	<0.001
Log likelihood ratio				<0.001

OR, odds ratio; CI, confidence interval.

Adjusted for sex, age, hypertension, dyslipidemia, diabetes, hypertension medications, diabetes medications, dyslipidemia medications, SBP, DBP, BMI, FPG, TC, LDL-C, and HbA1c.
